# Heart rate in professional musicians

**DOI:** 10.1186/1745-6673-3-16

**Published:** 2008-07-25

**Authors:** Claudia Iñesta, Nicolás Terrados, Daniel García, José A Pérez

**Affiliations:** 1 Departamento de Biología Funcional, Universidad de Oviedo and Unidad Regional de Medicina Deportiva del Principado de Asturias-Fundación Deportiva Municipal de Avilés, Spain; 2 Departamento de Biología de Organismos y Sistemas, Universidad de Oviedo, Spain; 3 Centro de Salud de Contrueces-Vega, Gijón, Spain

## Abstract

**Background:**

Very few studies have analysed heart rate (HR) with regard to music playing, and the scarce evidence available is controversial. The purpose of this study was to analyse the HR response of professional musicians during their real-work activity.

**Methods:**

Sixty-two voluntary professional musicians (20 women, 42 men), whose ages ranged between 15 and 71 years old, underwent the test while playing their instruments in real life scenarios, i.e. rehearsals, practice and public concerts. The musicians carried Sport Tester PE4000 (Polar^®^, Finland) pulsometers to record their HR.

In order to compare data from differently aged subjects we calculated their Maximum Theoretical Heart Rate (MTHR). Later on we found out the MTHR percentages (%MTHR) corresponding to the registered HR of each subject in different situations. The value of the MTHR for every musician was obtained by means of the *220 – age (in years) *formula.

**Results:**

Throughout the HR recordings, we have observed that musicians present a heightened HR while playing (in soloists, mean and maximum HR were 72% and 85%MTHR, respectively). Cardiac demand is significantly higher in concerts than in rehearsals while performing the same musical piece. The HR curves corresponding to the same musician playing in repeated concerts (with the same programme) were similar.

**Conclusion:**

The cardiac demand of a professional instrument player is higher than previously described, much greater than what would be expected from a supposedly sedentary activity.

## Background

The activities of professional musicians, be they rehearsals or public performances, have not been properly studied despite their social importance.

When studying the actual effort displayed by a musician while doing his/her work, it is necessary to find a reliable method which does not interfere with their artistic activity. Such a method should be accepted by the person under study, yielding reproducible and easily achievable data, besides being considered as valid by the scientific community. It is well known that, for at least the past 20 years, heart rate (HR) has been analysed and used to measure physical effort in the working and sports fields [1-4].

Heart rate can be modified by several environmental factors (temperature, moisture, atmospheric pressure, time of the day, height, adaptation level, noise), or physiologic ones (age, sex, digestion, health state), as well as those related to the activity itself (physical and mental compounds, grade of fitness or adaptation to the task, position, length of the activity, the fact of being under social evaluation) [1]. Despite all these influences, the continuous recording of HR truthfully mirrors the physical workload a given task implies. HR recordings obtained this way can be quantitatively and visually analysed, which allows to dynamically evaluate the circulatory load imposed by workloads with variable intensities [1-4].

Back in 1985, Åstrand and Rodahl proposed the classification of physical work based on HR reaction as shown in Table 1. Their data referred to average 20- to 30-year old subjects. Subsequently, the American College of Sports Medicine (ACSM) [5] published some recommendations about the amount of exercise needed to maintain cardiorespiratory fitness considering the age of the individuals, and classified the effort intensity level according to percentages of the Maximum Theoretical Heart Rate (%MTHR) reached during the exercise (Table 2). A subject's MTHR is the value obtained using the "220-age" (in years) formula, which is still considered as valid in spite of current controversies regarding its accuracy [6].

**Table 1:** Classification of prolonged physical work related to HR reaction, according to Åstrand and Rodahl.

Intensity of the effort	HR (bpm)
Mild work	Up to 90
Moderate work	90–110
Heavy work	110–130
Very heavy work	130–150
Extremely heavy work	150–170

**Table 2:** Intensities of physical work related to %MTHR, following the ACSM classification.

Intensity of the effort	%MTHR
Very light	< 35%
Light	35–54%
Moderate	55–69%
Hard	70–89%
Very hard	≥ 90%
Maximum	100%

What do we actually know about the professional musicians' work?. How are their tasks considered?.

Several guides inform about the energy expenditure (EE) of different jobs, leisure time and sportive activities. Only three of them include data on EE expressed in MET (basal metabolic unit) about musical activities [7-10]. These authors do not fully explain how their data were obtained to build their tables (Table 3).

**Table 3:** Energy expenditure (METs) depending on the different musical instruments played, according to different authors.

Instrument	McArdle	Ainsworth	Fletcher
Accordion	2.1	1.8	1.8
Drums	4.3	4	
Cello	2.7	2	2.3
Flute	2.3	2	2
Horn	1.9	2	1.7
Piano	2.6	2.5	2.3
Trumpet (standing)	2	2.5	1.8
Violin	2.9	2.5	2.6
Woodwinds	2.1	2	1.8
Writing, sitting	1.9	1.8	1.7
Walking (3.5 km/h)	2.7	2	2.5

When comparing the EE in these guides, typing (1.8 METs), or walking at 2 miles per hour (mph) (2 METs) are equivalent to the act of playing an instrument (see Table 3). Playing drums is the only activity considered as "more demanding" (4 METs) [9].

Are these data accurate?.

Until now, continuous HR recording as a tool for effort measurement has not been used in musicians. Various authors have carried out studies with other purposes on musicians using HR recordings. In 1964, Bouhuys [11] investigated the respiratory function of wind instrument musicians by means of a laboratory study which included HR measurements. Mulcahy (1990) [12] carried out the 24-hour HR recording of a group of professional musicians belonging to the BBC Symphonic Orchestra and members of the staff team, in order to show the need to adjust a reliable cardiovascular treatment to the daily working schedule. His purpose was to tailor treatments for optimal protection in patients with coronary artery diseases, taking into account the timing of occupational-induced changes in heart rate. Hunsaker (1994) [13] published a study about HR and cardiac rhythm responses in trumpet players using Holter monitors.

Due to a lack of scientific research about the efforts shown by musicians during their job, the aim of our study was to measure the HR of professional musicians while working, that is, during rehearsals and public concerts; to compare the obtained HR with the MTHR of each subject; and to evaluate the differences in cardiac demand in diverse work scenarios.

## Methods

Sixty-two subjects (20 women and 42 men), whose ages ranged between 15 and 71 years old, volunteered to take part in this study. They were members of the main orchestras, as well as teachers and advanced students of the Conservatories of the Princedom of Asturias (Spain).

**Figure 1:**
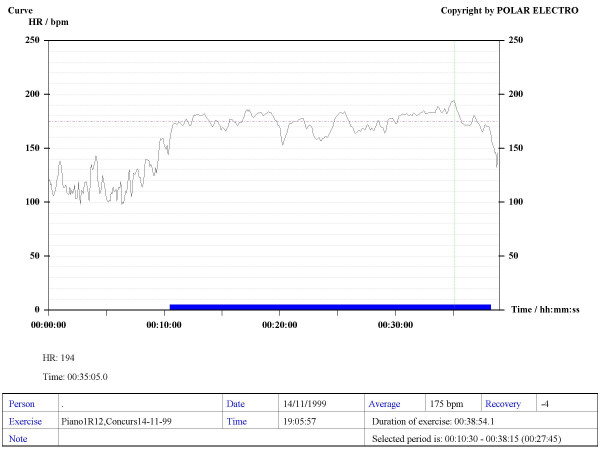
**Recording corresponding to a pianist during an important performance**. The MHR throughout the concert was 175 bpm, with a Max HR of 194 bpm, while performing Bartok's *14^th ^suite*.

**Figure 2:**
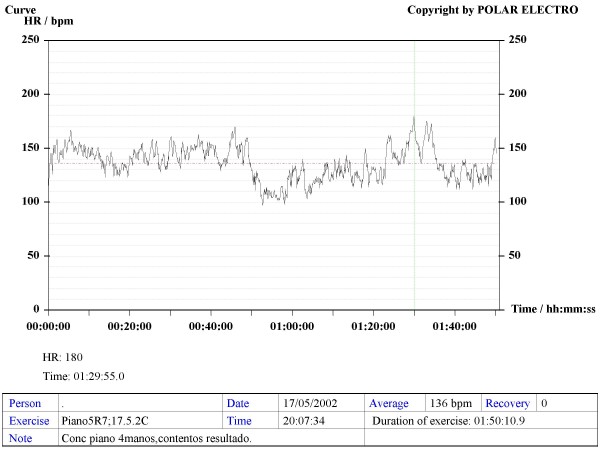
**HR recording of an acknowledged 41-year-old pianist during a four-hand piano concert.** She maintains a 136 bpm MHR for almost two hours. During part of the programme her HR goes over 150 bpm, reaching 180 bpm Max HR which, for this subject, means a 101% of her MTHR.

**Figure 3:**
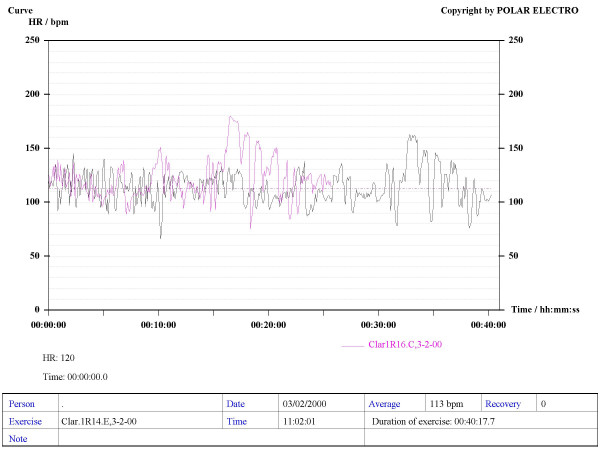
**Overlapped printouts belonging to the main clarinetist of a symphonic orchestra during the REHEARSAL (in black ink) and the CONCERT (pink line) of the same musical piece**. The rehearsal time is longer, due to the conductor's explanations.

**Figure 4:**
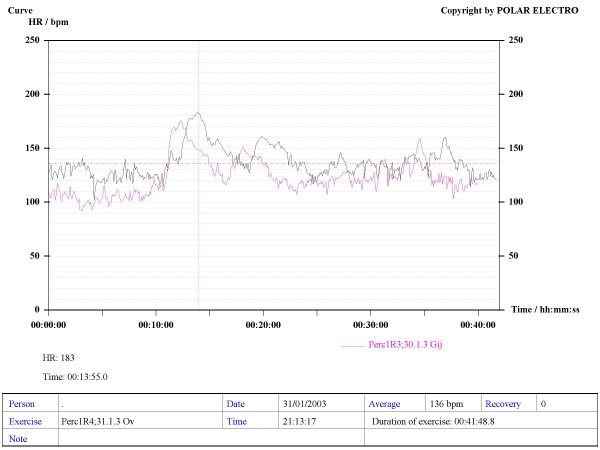
**An orchestral percussionist performs two concerts in two different days, playing the same programme (C1 – C2)**. The pink line corresponds to the first concert. The MHR is 136 bpm, and the Max HR is 183 bpm.

**Figure 5:**
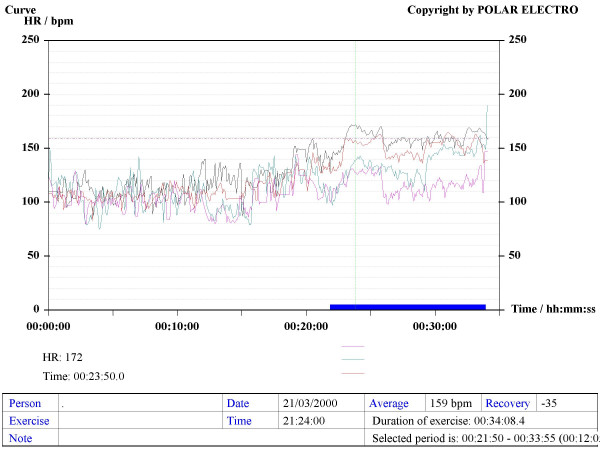
**The components of a string *quartet *(first and second violins, viola, cello) record their HRs.** Before the concert begins (around the 22^nd ^minute of the recording) their HRs are not too different, but when it starts, the graph displays different lines, according to the different roles throughout the performance.

**Figure 6:**
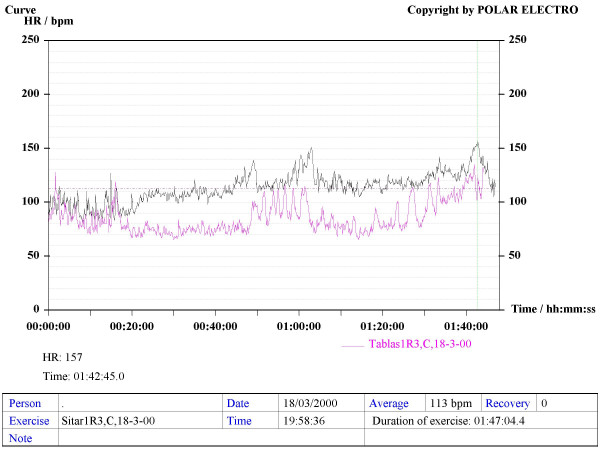
*Sitar *and *tabla *duet. The concert starts at the 15^th ^minute of the recording with a long *sitar *introduction called *Alap*, a slow part with no defined rhythm. The *tabla *player keeps a respectful silence throughout that introductory part and, from the 49^th ^minute of the recording onwards, he joins the *sitar *player performing increasingly complex and fast sequencial pieces.

Our method consisted in using Sport Tester PE4000 (Polar^®^, Finland) devices programmed to record a cardiac frequency value every five seconds. The musicians were trained to use the devices to record their HR during rehearsals and concerts. The average duration of concerts and rehearsals was one hour. A graphic, printable curve allowed us to analyse the course of the HR in each recording scenario as well as the Maximum (Max HR), Mean (MHR) and Minimum HR values, plus the date and time of the day when the recording was made (see various examples in Figures 1 to 6). On the printouts, the blue bar indicates the recording time that corresponds to the musical performance. The data obtained were uploaded to a personal computer for further analysis by means of the Polar Advantage Interface System.

The subjects were classified into five instrumental groups: strings, winds, piano, percussion and classical Indian music players (Table 4).

**Table 4:** Distribution of subjects according to the different scenarios where the recordings took place and the instruments they played.

Instrumental group	Subjects	Recordings	Rehearsals	Concerts
Winds	25	209	79	130
Strings	23	130	55	75
Piano	10	89	43	46
Percussion	2	19	-	19
Classical Music of India	2	5	-	5

509 registers were obtained, out of which 452 were determined as valid for further analysis. Those showing interferences between pulsometers, disconnection mistakes due to excessive distance between the chest belt sensor and the wrist receptor, or badly adjusted sensors were excluded. The higher number of registers analysed corresponded to the winds and strings groups, since they are also the most representative and numerous in an orchestra.

All musicians work in a sitting position, although percussion players and some soloists play in a standing position.

Fifteen members of the study underwent a medical exercise test in a cycle-ergometer until exhaustion, in order to find out their Real Maximum HR, and compare it to the Maximum Theoretical HR (MTHR).

Eight subjects registered their Basal HR in the morning just as they woke up in bed, before getting up.

### Statistical analysis

The purpose of the statistical analysis was to verify whether there were any significant differences in %MTHR, MHR and Max HR values (dependent variables) across the different types of activity.

As a prior step, in order to test whether the dependent variables adjusted to a normal distribution, the Shapiro-Wilk test was carried out. The sample comprised the pooled data of concerts from the winds and strings groups. As a result, we found out that %MTHR for MHR and Max HR showed distributions far different from what would be considered a normal one, and thus we chose non-parametrical statistics (Wilcoxon test for paired samples).

Spearman rank correlation tests were performed to explore how stable the percentages of MTHR (for Mean HR and Max HR values) were among individuals, across different performance scenarios.

Recordings of different musical works performed by the same musician cannot be considered as being statistically independent samples (*pooling fallacy*) [14,15]. Thus, the units considered for analysis were individual musicians, and not musical pieces, in order to avoid pseudoreplication [14,15]. In order to do so, we pooled all played musical works for each different musician and considered the average value of the measured variables (Mean and Maximum HR and their corresponding %MTHR).

The types of musical activities to compare were:

1) REHEARSAL *versus *PUBLIC CONCERT of the same musical pieces performed by the same subject;

2) FIRST CONCERT (C1) *versus *SECOND CONCERT (C2), in which a given subject recorded his or her HR while playing the same musical pieces in two different public concerts.

The Wilcoxon Test for paired samples was used to make the statistical comparisons, and comparing the Real Max HR with the MTHR in those subjects who underwent the effort test.

## Results and Discussion

Tables 5 and 6 show the values of the averages and (±) standard deviations (SD) of Max HR and MHR, as well as their corresponding %MTHR, belonging to the HR registered during Rehearsals and Concerts of the same musical pieces, performed by different instrumental groups.

**Table 5:** Max HR and MHR values (bpm), with their corresponding %MTHR in Rehearsal and Concert scenarios.

	Rehearsal			
Instrumental group	Max HR	%MTHR	MHR	%MTHR

WINDS	132 ± 17	68 ± 9	101 ± 13	52 ± 7
STRINGS	117 ± 14	62 ± 8	89 ± 15	47 ± 7
PIANO	116 ± 19	61 ± 13	93 ± 15	49 ± 10

	Concert			

Instrumental group	Max HR	%MTHR	MHR	%MTHR

WINDS	151 ± 18	79 ± 10	118 ± 23	61 ± 11
STRINGS	137 ± 23	72 ± 10	110 ± 26	57 ± 12
PIANO	167 ± 20	86 ± 13	140 ± 16	72 ± 9
PERCUSSION	149	81	108	59
Musicians from INDIA	161	86	105	56

**Table 6:** Max HR and MHR values (bpm) with their corresponding %MTHR, in musicians performing as SOLOISTS.

Instrumental group	Max HR	%MTHR	MHR	%MTHR
WINDS	167 ± 15	87 ± 7	139 ± 18	73 ± 9
STRINGS	164 ± 14	82 ± 7	142 ± 19	71 ± 9
PIANO	167 ± 20	86 ± 13	140 ± 16	72 ± 9

Average values are important from an analytical point of view in order to contrast hypotheses, but they can mask the biological aspect of measurements, which is "contained" within standard deviation values.

On Table 5 we can observe how, even in a REHEARSAL scenario, the average values of Max HR are over 115 bpm. This was the highest value found by Bouhuys [11] in a laboratory study which consisted of playing music for five to seven minutes, leading him to classify this effort as "less than heavy". Although the musical piece played included a wide range of notes and expressive notations, it was nonetheless a laboratory test.

In the CONCERT scenario, the average values of Max HR range from 137 bpm in the strings group to 167 bpm in the pianists'. These values could be classified as "heavy" and "very heavy" according to the intensity levels of effort (Tables 1 and 2). Mean HR is, however, even more relevant than Max HR, since its values reveal the intensity of the sustained effort during each concert, all placed in our data between the "mild" and "heavy" or "hard" levels (Tables 1 and 2).

In the case of SOLOISTS (Table 6) the demanded effort is even more evident, since MHR values are 139 ± 18 bpm (winds), 142 ± 19 bpm (strings), and 140 ± 16 bpm (piano), whereas Max HR values are 167 ± 15, 164 ± 14, 167 ± 20 bpm respectively during concerts. According to Åstrand and Rodahl [1] (Table 1), these HR values could correspond to intensity levels ranging between "heavy" and "very heavy". Based upon the ACSM classification (Table 2), these %MTHR in concerts stand for a "heavy" level of work intensity [5].

**Figure 7:**
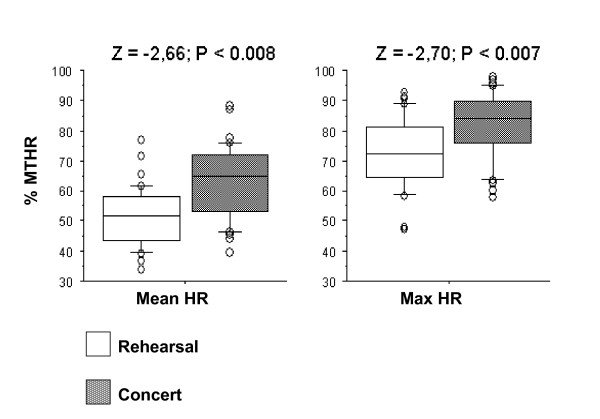
**Wind instruments: Rehearsal-Concert comparison**. Box-plot representing the distribution of the %MTHR of the MHR and Max HR values according to Rehearsal or Concert scenarios.

**Figure 8:**
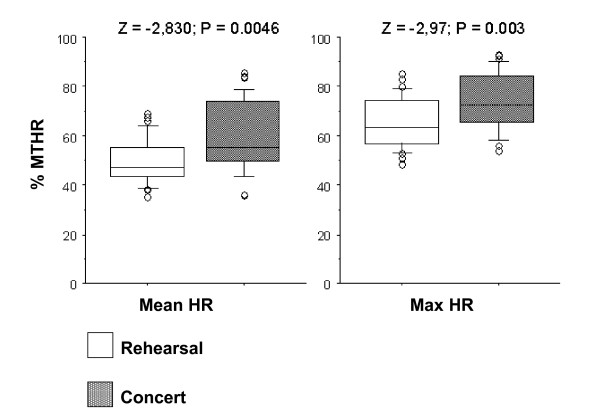
**String instruments: Rehearsal-Concert comparison**. Box-plot representing the distribution of the %MTHR of the MHR and Max HR values according to Rehearsal or Concert scenarios.

**Figure 9:**
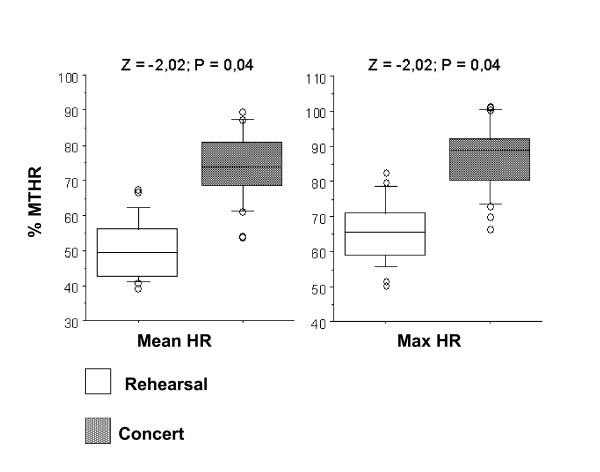
**Piano: Rehearsal-Concert comparison**. Box-plot representing the distribution of the %MTHR of the MHR and Max HR values according to Rehearsal or Concert scenarios.

**Figure 10:**
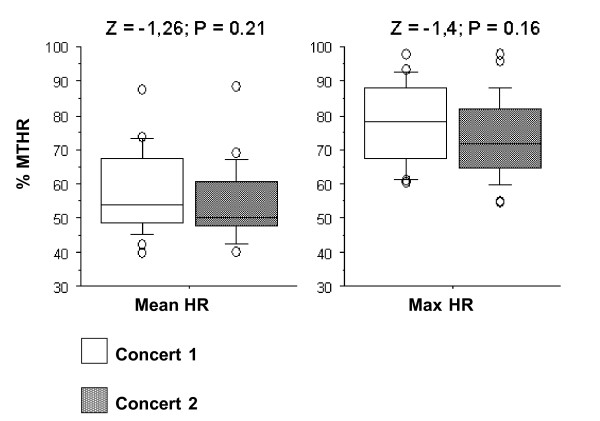
**Wind instruments: Concert 1-Concert 2 comparison**. Box-plot representing the distribution of the %MTHR of the MHR and Max HR values according to C1 *versus *C2 scenarios.

**Figure 11:**
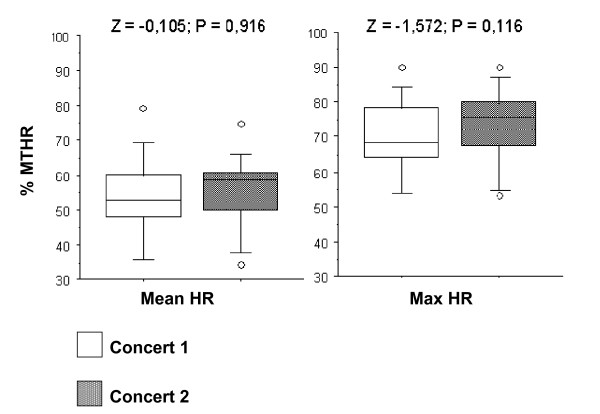
**String instruments: Concert 1-Concert 2 comparison**. Box-plot representing the distribution of the %MTHR of the MHR and Max HR values according to C1 *versus *C2 scenarios.

In the Box-Plots figures (Figures 7 to 11) we show the %MTHR distributions corresponding to MHR and Max HR achieved by the musicians, depending on the analysed scenario. The horizontal inner line represents the *median *value of the collected data. The box itself contains the middle 50% of the data, settled down between the 25^th ^and 75^th ^percentiles. The so-called "whiskers" are the 5% and 95% limits, and the external circles or points are the outliers or extreme values of the distribution. The results of a Wilcoxon test (Z-value and level of significance) comparing REHEARSAL and CONCERT or CONCERT 1-CONCERT 2 scenarios are also shown for each dependent variable (MHR and Max HR) at the top of the Figures.

All figures presented *median*, 25% and 75% quartiles, and 5% and 95% percentile values lower in the REHEARSAL scenario than in the CONCERT scenario.

Based on these results, HR is significantly more demanding in the CONCERT scenario than the REHEARSAL scenario in the winds, strings and piano groups (Figures 7, 8 and 9).

This difference was already hinted at by the results of Mulcahy and Hunsaker studies [12,13] (carried out with other purposes [12], or based on only one type of instruments [13]). Mulcahy calculated the average of the pooled Max HR recorded from members of a symphonic orchestra (including management, technical staff and musicians who did not play for a great length of the programme). This could be the reason why the average Max HR were 91.3 bpm (rehearsal) and 97.7 bpm (concert), that is, lower than the values obtained in our study.

Hunsaker shows in one of her Tables the values of Mean HR recorded by nine trumpet players during a rehearsal and a public concert, performing the same musical piece. She carried out her study by means of Holter monitors. In eight subjects, Mean HR were higher during the concert, and more rhythm alterations in the EKG were detected. None of these alterations persisted once the performance was over. She concluded that these EKG changes could be considered as normal variants in otherwise healthy subjects, and they occur only when playing a musical instrument. In our study, we statistically demonstrate those HR differences in the winds, strings and piano groups. On the other hand, the Holter device could be unsuitable for musicians [4], especially during concerts.

When comparing the registered HR during two concerts performing the same musical programme, at the same time of the day in two different days (the so-called CONCERT 1-CONCERT 2 situation), we found no significant difference between them. This is true for winds and strings players (Figures 10 and 11). The HR curves for both scenarios overlap, which shows an almost identical cardiac effort when the musician performs the same programme. The repeatability of the obtained recordings can be observed, in addition to the reliability and their possible reproducibility (Figure 4).

It was not possible to make a statistical comparison between C1–C2 with neither piano players, percussionists nor classical Indian music players, because only two subjects got recordings in that situation. These two latter groups made recordings only in the CONCERT scenario.

The HR recordings of two Hindi musicians throughout their concerts (complete *ragas *which featured slow and fast tempos) showed a cardiac activity similar to that of Western classical musicians (Table 5, Figure 6), in spite of being a type of music with a demonstrated relaxing effect on cardiac frequency, at least on the part of the listener [16,17].

Besides the main result of this study, our empirical, comparative approach also highlights the need for out-of-laboratory measures in the study of cardiac effort. Abel and Larkin had observed different cardiovascular responses in laboratory *versus *natural settings, proving the lack of accuracy if data were extrapolated [18]. Larger and Ledoux acknowledge that "cardiovascular measurements in musicians should be procured, ideally, under actual working conditions at rehearsals, or during live public performance of music requiring greater and lesser degrees of mental and physical effort" [19].

According to the HR obtained in our study, it is surprising to find out that playing an instrument could be equivalent to writing while sitting in terms of energy expenditure, as previously described (Table 3).

More research would be necessary to further analyse the reasons why there exist differences between rehearsal and concert HR, since the subjects who took part in our study are professionals who perform their tasks without showing any symptom of stage fright or performance stress.

On the other hand, Clark and Agras, after successfully treating stage anxiety in musicians via cognitive-behavioral therapy, did not find the expected decrease in HR during musical performance [20].

Whichever is the cause, we have observed a significant increase in HR during concerts; hence, musicians, especially soloists, must be aware of this circumstance and be ready to face it not only with psychological coping techniques but also by undergoing an adequate physical conditioning.

### Exercise Test Results

The average age of the 15 subjects who underwent the medical exercise test was 31.2 ± 6.8 years old. The MTHR corresponding to this age is 188.8 ± 6.8 bpm, using the 220-age (in years) formula.

The average Max HR achieved during the exercise test in this group was 187.2 ± 11.9 bpm.

There were no statistical differences between the Real Max HR and the MTHR in this group of individuals (Wilcoxon test: Z = -0.341; p = 0.733 for N = 15 subjects).

The average Basal HR value of the 8 individuals who presented this data was 50 ± 9 bpm.

## Conclusion

Up to now, the study of pathologies in professional musicians has been almost exclusively focused on neuromuscular injuries and problems related to stage fright. This study reveals an unknown facet of the musical profession, as it objectively shows the cardiac effort that musicians must exert when performing. Our study describes a physiological response of professional musicians with clear implications on work health, and it links the variability of this response to the explicit gradients of professional activity.

Heart frequency is significantly higher in public concerts than in the rehearsals of a given musical piece. During public concerts, professional musicians as a group reach Mean HR of 60.2% of their MTHR. These musicians show average Max HR of 76.8% of their MTHR. These HR values are higher than previously described, and could be placed in the "moderate" to "heavy" levels of work intensity.

The Real Max HR studied in the subjects who carried out an exercise test by cycle-ergometer was statistically similar to their MTHR.

Physicians must be aware of the cardiac effort that a certain musician patient has to face when he or she goes back to work after a cardiovascular event. Musicians, especially soloists, must be aware of the energy surge their heart will need while performing in a concert, and must be ready for it both with psychological coping techniques and by undergoing an adequate physical conditioning.

Therefore, our findings encourage professional musicians to observe healthy life habits in order to prevent cardiovascular pathologies. We strongly recommend professional musicians to do regular physical exercise, since it enhances cardiovascular health, and boosts endorphin levels, which in turn heightens stress management and procures a sensation of well-being [21,22].

## List of abbreviations used

HR: Heart rate; bpm: beats per minute; EE: Energy expenditure; MET: Metabolic equivalent, (unit of resting oxygen uptake = 3.5 mL O_2 _per kilogram body weight per minute; mL O_2_. kg^-1^. min^-1^); MTHR: Maximum Theoretical Heart Rate; %MTHR: Percentage of MTHR; MHR: Mean HR; Max HR: Maximum HR; Av HR: Average HR; mph: Miles per hour; C1: First concert; C2: Second concert; ACSM: American College of Sports Medicine; EKG: Electrocardiogram; Km/h: Kilometres per hour; N: Number of individuals.

## Competing interests

The authors declare that they have no competing interests.

## Authors' contributions

CI and NT participated in the design of the study, revision of the medical literature, data collection, and drafted the manuscript. DG carried out the statistical analysis and helped to draft the manuscript. JAP helped to revise the medical literature and to draft the manuscript.

All authors have read and approved the final manuscript.
